# Preventing and Managing Chronic Disease Through Implementation Science: Editor’s Introduction to the Supplemental Issue

**DOI:** 10.1007/s11121-023-01617-y

**Published:** 2023-12-01

**Authors:** Justin D. Smith, Sandra F. Naoom, Lisa Saldana, Sharada Shantharam, Tina Anderson Smith, Jennifer M. Kohr

**Affiliations:** 1 Department of Population Health Sciences, Division of Health Systems Innovation and Research, Spencer Fox Eccles School of Medicine at the University of Utah, 295 Chipeta Way, 84108 Salt Lake City, UT USA; 2 JSFB LLC, Northbrook, IL USA; 3 Chestnut Health Systems, Lighthouse Institute, Eugene, OR USA; 4 Centers for Disease Control and Prevention, Division for Heart Disease and Stroke Prevention, Atlanta, GA USA; 5 Anderson Smith Consulting, Atlanta, GA USA; 6 Centers for Disease Control and Prevention, Performance and Evaluation Office, Atlanta, GA USA

## Introduction

People living with cardiovascular disease and other chronic conditions had a greater risk of complications and death during the COVID-19 pandemic (Abbasi, 2022; Clerkin et al., 2020; Vosko et al., 2023; Xie et al., 2022). Like many other health conditions, chronic diseases disproportionately affect people from minority groups and people with lower incomes (Caraballo et al., 2022; Crook & Peters, 2008). These health disparities were exacerbated by the COVID-19 disease and the effects of pandemic response measures on preventive healthcare in the USA (Andraska et al., 2021; Boehmer et al., 2022; Lopez et al., 2021). Amid the unprecedented public health crisis of COVID-19, there were many opportunities for prevention and for implementation scientists to create and test innovative solutions to mitigate these effects (Wensing et al., 2020).

Implementation science has emerged as a potential solution to the failure to translate evidence from research into effective practice (Eccles & Mittman, 2006) and policy evident in many fields. Implementation science in health is the study of methods to promote the adoption and integration of evidence-based practices, interventions, and policies into routine healthcare and public health settings to improve our impact on population health (National Institutes of Health, 2022). The field seeks to understand the approaches that work best to translate research to real-world systems of care and further apply and adapt these approaches in different contexts and settings to improve public health. Implementation science, thus, could help maximize reach and impact of interventions for populations with chronic diseases.

This supplemental issue of *Prevention Science* titled, *Advancing the Adaptability of Chronic Disease Prevention and Management Through Implementation Science*, brings together contributions by researchers and practitioners in the fields of chronic disease prevention and implementation science. The overall objective of this supplemental issue is to examine the intersection of chronic disease prevention and management with implementation science, with the goals of (a) providing a resource for public health researchers, public health practitioners, government officials, and other decision-makers and (b) sharing lessons learned that can inform infrastructure development for the dissemination and implementation of evidence-based public health interventions. In this Editor’s Introduction, we build on the work of others (e.g., Chambers, 2018; Estabrooks et al., 2018) in describing a novel perspective that could be used by national public health organizations and local health departments to place implementation science within the Research to Practice continuum to maximize public health impact of evidence-based preventive interventions (EBPIs). The COVID-19 pandemic both contributed to and underscored significant health disparities in chronic diseases in the USA. This supplemental issue also addresses ways in which implementation science holds promise to address disparities and achieve health equity across diverse communities and individuals. We then illustrate how articles in this supplemental issue align with this perspective and the overall objective and goals of the supplement.

### Effective Practice Pipeline

**Fig. 1:**
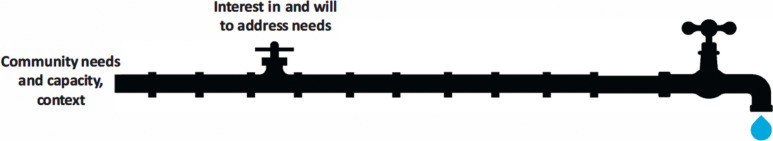
Effective practice pipeline: community pipeline

**Fig. 2:**
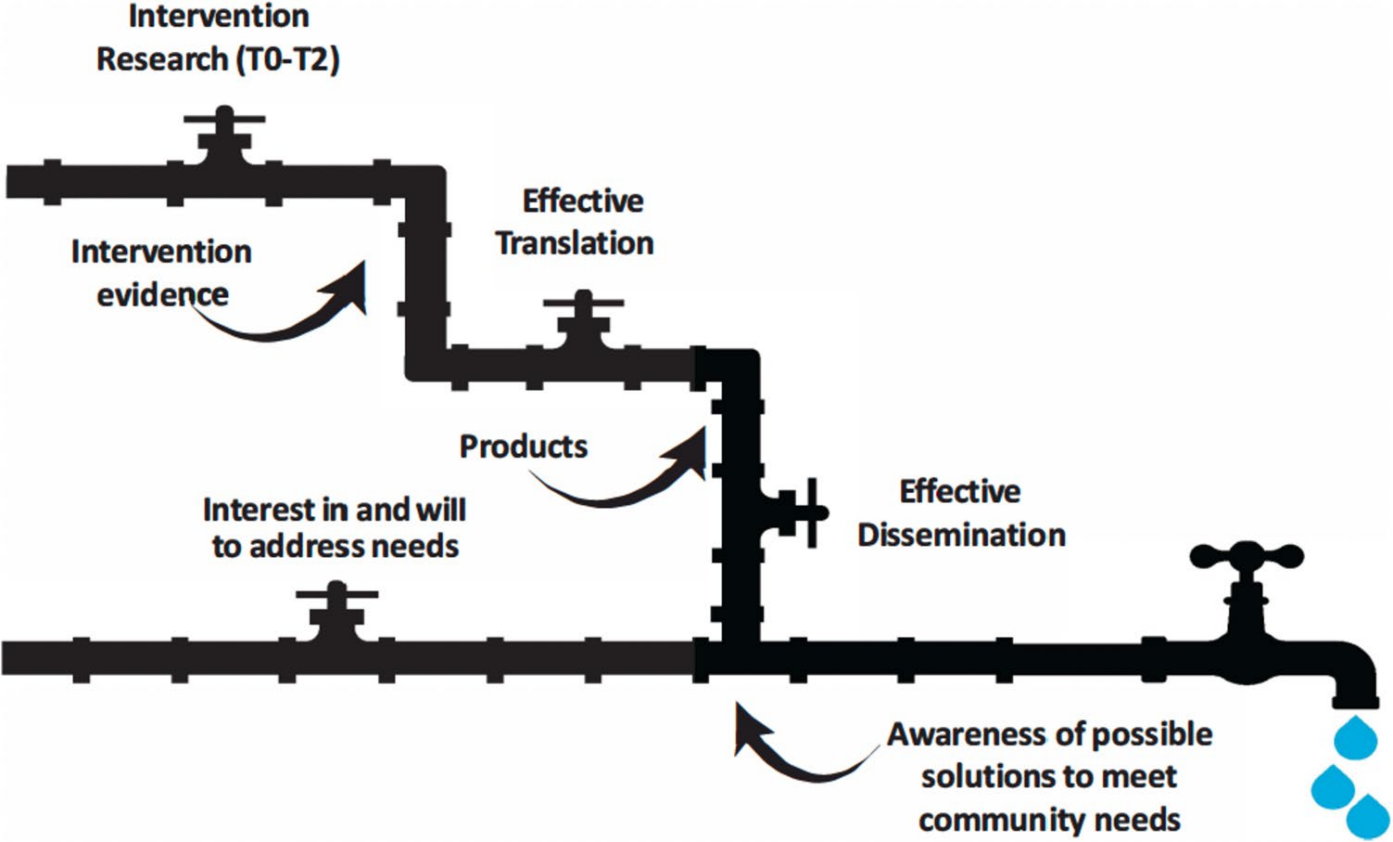
Effective practice pipeline: intervention research pipeline

**Fig. 3:**
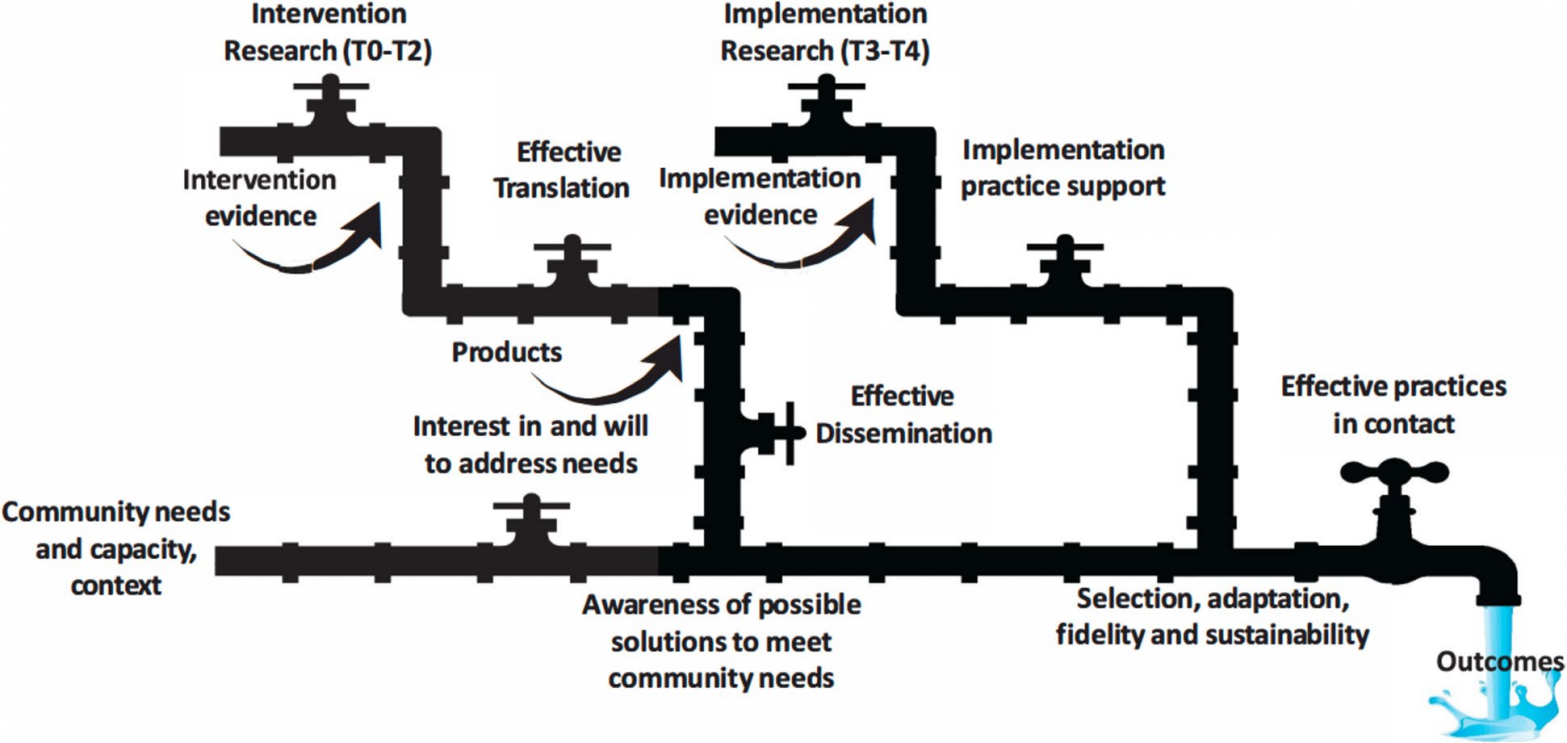
Effective practice pipeline: implementation research and implementation practice pipeline

**Fig. 4:**
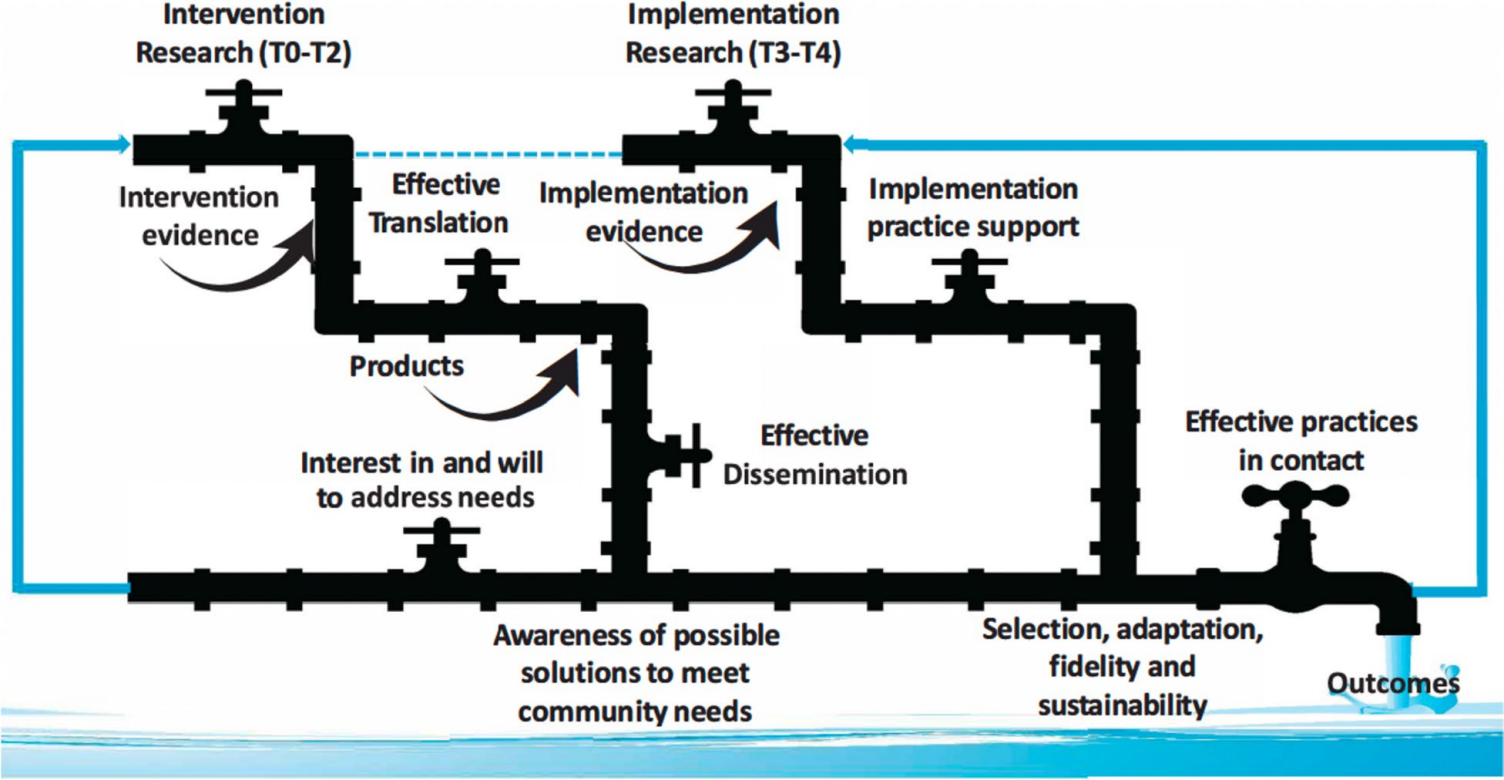
Effective practice pipeline

Three of the authors (Naoom et al., 2019) developed the Effective Practice Pipeline to show how implementation science is made up of research (the evaluation of strategies to increase the adoption, reach, and sustainment of EBPIs) and implementation practice support (resources and capacity building on the use of strategies by public health agencies and practitioners). Both are needed to generate public health impact. The “pipeline” visual, introduced in detail next and divided into three figures (Figs. 1, 2, and 3) that together form the full pipeline (Fig. 4), was chosen to show how using effective implementation science can increase the flow toward effective practice in context for diverse communities. The Effective Practice Pipeline was intended to specifically situate the use of implementation research by practice sites within the more general research-to-practice continuum. It was informed in part by translational science models, such as the National Center for Advancing Translational Science of the National Institutes of Health’s Translational Science Spectrum (National Center for Advancing Translational Science, 2021) and by frameworks developed by the National Implementation Research Network (Fixsen et al., 2005).

### Community Pipeline

Ultimately, public health science aims to promote and protect the health of all populations (American Public Health Association, 2022). The Effective Practice Pipeline begins with the people and communities, in the broadest possible sense, served by public health entities. The community pipeline illustrated in Fig. 1 shows the connection between communities’ varying needs, capacities, resources, and contexts to address health-related needs. The rate of intervening on these needs is influenced by the community’s interests and will to address them (depicted as the spigot). Some communities will open the spigot on the pipeline and begin to see a trickle of salient outcomes on their own as shown in Fig. 1, whereas others might desire a greater flow. The capacity of the community to obtain greater flow can be greatly improved by implementation science when it is effectively translated to implementation practice nationally and locally.

### Intervention Research Pipeline

Communities may struggle to achieve meaningful flow through the pipeline for several reasons, ranging from a lack of awareness of which EBPIs will meet their needs to a lack of technical knowledge of the best strategies to implement for their context, given resource constraints. Figure 2 introduces the intervention research pipeline to the previously described community pipeline. Intervention research produces evidence or best practices that can be translated into products including programs, policies, guidelines, and technical packages that are disseminated among specific populations and/or communities. Intervention research has been characterized along the Translational Science Spectrum (National Center for Advancing Translational Science, 2021) as being Translational (T) Stage T0 (Basic Research—fundamental mechanisms of biology, disease, or behaviors); T1 (Preclinical Research—scientists develop model interventions to further understand the basis of a disease or disorder and find ways to treat it); and T2 (Clinical Research—studies to better understand a disease in humans and relate this knowledge to findings in cell or animal models; testing and refinement of new technologies in people; testing of interventions for safety and effectiveness in those with or without disease; behavioral and observational studies; and outcomes and health services research). The result of moving through Stage T2 with positive clinical outcomes is evidence for specific interventions, programs, and practices (i.e., EBPIs).

Several repositories and clearinghouses compile and evaluate the evidence for specific preventive interventions to inform potential adopters of their suitability to meet community needs, such as CDC’s Best Practices Clearinghouse for Heart Disease and Stroke Prevention and Management (https://hdsbpc.cdc.gov/s/) and Blueprints for Healthy Youth Development (https://www.blueprintsprograms.org/). Such registries increase decision-maker and implementer awareness of the possible solutions to their communities’ challenges. However, program awareness and interest are insufficient for the successful implementation of new EBPIs; awareness must be coupled with support to select the best possible solution, effectively tailor it for the community, and develop the infrastructure and necessary supports to put it in place with fidelity and sustainment (Alley et al., 2023)—the purview of implementation research.

### Implementation Research Pipeline

As noted previously, implementation science includes two elements: research and practice. Implementation research seeks to understand the approaches that work best to translate research to the real world. Implementation practice seeks to apply and adapt these approaches in different contexts and settings to achieve outcomes (Ramaswamy et al., 2019). Implementation research aligns with Stages T3 (Clinical Implementation—the adoption of interventions that have been demonstrated to be useful in a research environment into routine clinical care for the general population) and T4 (Public Health—researchers study health outcomes at the population level to determine the effects of diseases and efforts to prevent, diagnose, and treat them). Figure 3 highlights that like intervention research, implementation research produces evidence in the form of models, frameworks, and strategies that when utilized as prescribed, contribute to effective practice and the achievement of salient implementation outcomes. Implementation outcomes are the direct effects of implementation and include, but are not limited to, adoption of the EBPI, reach to the intended population, program delivery cost, fidelity of delivery, and sustainment of the EBPI over time. Such implementation outcomes contribute to the overall public health impact of the EBPI in reducing the burden of chronic disease for individuals and communities. The implementation research pipeline in Fig. 3 demonstrates that evidence generated by rigorous research makes effective implementation practice support possible.

Numerous organizations and individuals are needed to support implementation through the utilization of implementation research evidence. These include public health departments, technical assistance providers, and the individual providers who ultimately deliver EBPIs. This requires an operationalization of what it takes—strategies and resources—to implement EBPIs and clear guidance on the enactment of the strategies shown to be effective in implementation research. Although the specific implementation tasks needed to implement a program might be tailored to that EBPI (e.g., training lay staff in blood pressure monitoring; training Masters level counselors in behavioral assessment), there is a common set of general implementation strategies (e.g., training frontline staff) utilized across EBIs (Wong et al., 2022). Previous research (Alley et al., 2023) has identified forty-six strategies commonly utilized across EBI implementations and have been operationalized using the Universal Stages of Implementation Completion (UniSIC). The UniSIC defines implementation tasks along an 8-staged process: (1) Engagement, (2) Consideration of Feasibility, (3) Readiness Planning, (4) Hiring and Training, (5) Establishing Fidelity Monitoring, (6) Program Launch, (7) Ongoing QA Monitoring, and (8) Competency in Delivery (Singh & Saldana, 2022). These strategies span the Implementation Research Pipeline (Fig. 3) and include use of evidence, practice support, and effective alignment with context to increase the flow of positive outcomes for communities.

Achieving the promise of the vast investment in developing and testing EBPIs requires all three pipelines to be married and to operate synergistically. This complex process requiring multiple feedback loops is shown in the complete Effective Practice Pipeline in Fig. 4. As the Translational Science Spectrum moves from T0 (basic research) to T4 (efficacy research), both encompass intervention and implementation research. What we learn in the earlier phases—T0 through T2—influences phases T3 and T4. Though similar to the iterative process of implementation, it also is possible that what we learn when conducting implementation research will inform future intervention research for that particular EBPI or for researchers developing other EBPI’s. This bidirectional feedback loop is represented in Fig. 4 by the dotted line. The arrows in Fig. 4 that flow from the bottom to the top represent the practice-to-research feedback loops.

There are lessons that can be learned from the implementers shepherding the implementation of an EBPI in a community that can both inform the intervention characteristics and implementation strategies and processes. These actors are in a position to provide valuable insights on the barriers that exist to adoption, fidelity, reach, and sustainability. Additionally, engaging and heeding the voices and concerns of community members for whom these interventions have been designed can inform the entire research enterprise—intervention developers, implementation scientists, and implementation practitioners—enhancing chronic disease prevention and management and advancing health equity (Mensah et al., 2018; Philbin et al., in press).

Intervention research and implementation pipelines that are conducted in service to the community pipeline may help achieve benefits for communities and the public health of the nation (Rudd et al., 2020; Glasgow et al., 2003). Additionally, evidence that is generated also needs to be useful to the community and aligned with their needs and interests. Building implementation practice capacity is the next crucial element for realizing the potential of the Effective Practice Pipeline.

## Building Implementation Practice Capacity

As the field of implementation science continues to grow, there is an increasing urgency to build implementation practice capacity, which currently lags far behind implementation research capacity. Estabrooks et al. (2018) called for the advancement of individual and team-based skills to build and sustain opportunities for implementation science to achieve public health outcomes. Implementation practitioners are professionals who support organizations, leaders, and staff in their implementation of evidence-informed practices and policies (Metz et al., 2017). They identify, contextualize, and improve the use of evidenced-informed implementation strategies in a range of settings. They can be referred to as coaches, improvement advisors, technical assistance providers, facilitators, consultants, mentors, and implementation specialists. Implementation practitioners often reside outside of the service delivery systems (e.g., healthcare, schools, community-based organizations) and instead work for intermediary organizations. But they may also reside within a service delivery system when those systems have work units specifically designed to support implementation and scaling efforts. Ideally, implementation practitioners attain competency in skillfully applying strategies to support adoption and sustained delivery with fidelity of EBPIs. Skillful application means deciding which strategies to put in place, given contextual factors and available resources.

Public health entities can help support implementation practitioners by providing comprehensive guidance on the strategies needed to implement specific EBPIs and how these strategies are intended to overcome barriers to implementation. Public health entities are uniquely situated to have a comprehensive understanding of the resource limitations of communities and the areas that are most in need of intervention. Such entities often are the first to detect communicable disease outbreaks (e.g., COVID-19) and chronic disease population effects (e.g., high incidence asthma) and identify community need.

Practitioners also need capacity and support to evaluate their implementations to understand which strategies are working to achieve specific implementation outcomes. Tracking implementation strategy use over time can lead to a more nuanced understanding of what it takes to implement different EBPIs, the types of strategies that are most useful during specific phases of implementation, and how implementation strategies need to be adaptively applied throughout the course of a given initiative, as contextual factors change, and new barriers and facilitators of implementation emerge and recede. Rigorous research and evaluation of implementation in action is critical to understanding the mechanisms of how strategies operate in specific contexts to effect implementation outcomes. Tucker-Brown et al. (2023) present a rigorous mixed methods evaluation, adapted from an established methodology developed by Leviton and Gutman (2010), of the implementation of the Hypertension Management Program in a federally qualified health center. The evaluation included a review of program documents, qualitative interviews, and micro-costing to evaluate implementation processes, facilitators and barriers, and the values of resources used to implement the program. The mixed methods analysis was critical to revealing relationships among these different data sources and providing a more complete picture of the implementation, including potential mechanisms, and what it would take to scale up the Hypertension Management Program in other federally qualified health centers. Understanding mechanisms is critically important to replicating findings, learning from negative studies, or adapting a strategy developed in one setting to another. The mechanisms through which strategies influence implementation outcomes are crucial for informing ongoing modifications to strategies and their use (e.g., they could be discontinued if ineffective). Without understanding implementation mechanisms, it is difficult to design strategies to produce expected effects across contexts.

## Population Benefit of EBPIs

The promise of implementation science has yet to be fully realized due to the numerous places in the Effective Practice Pipeline where spigots can fail to be opened or are opened only enough to allow a trickle. When the Community, Intervention, and Implementation Pipelines all flow together, a stream of necessary implementation outcomes (e.g., reach to the intended population) can be realized to improve public health at scale. Using the RE-AIM implementation evaluation framework, Gaglio et al. (2013) illustrated how population benefit of an EBPI is the collective effects of implementation outcomes: Adopting EBPIs and delivering them with fidelity to reach the intended recipients in a sustainable way are critical for overall population benefit. Implementation strategies are used to facilitate achievement of these outcomes.

Within this special supplement, Kohn et al. (2023) examined the feasibility of remote evidence-based fall prevention programs among older adults with disabilities. Using the RE-AIM framework, they found that adapting in-person programs to remote delivery was not only feasible but also accepted by community organizations and leaders. They also found the program to be feasible in terms of program delivery cost. Lovan et al.’s (2023) article examined the impact of implementation processes, cultural processes, and individual family factors in supporting Hispanic adolescents with unhealthy weight. Each of these factors played an important role in improving the adolescents’ quality of life, physical activity, and body mass index.

## Health Equity and Implementation Science

The field of implementation science recently has increased the emphasis on finding solutions to how implementation itself (the way EBPIs are delivered) contributes to and can help to alleviate health disparities with an explicit goal of using implementation research methods and strategies to achieve health equity. Scholars have recognized that implementation science can exacerbate health disparities if its use is biased toward implementing systems and communities that already have the highest capacities for delivering EBPIs (McNulty et al., 2019). Implementation science affords opportunities to iterate upon observed indicators of health disparities (e.g., reach to certain individuals/populations is lacking compared to others; effects of the intervention appear to be impacted by the presence of social determinants and health-related social risks for certain individuals/populations; implementation is inequitable for some implementing public health entities) (e.g., Kho et al., 2022). In cases of observed disparities that could be attributed to implementation, adaptive study designs (Curran et al., 2023) coupled with ongoing monitoring and improvement methods provide rigorous means of adapting implementation strategies aimed at building capacity to overcome context- and population-specific challenges.

A commentary by Shelton and Brownson (*this issue*) provides a detailed overview of contemporary thinking and suggestions for how implementation researchers and practitioners can make use of existing methods and research evidence to achieve health equity. Other articles in the supplement provide methods or findings related to implementation science and health equity for chronic disease conditions. Jacobs et al. (2023) describe how they integrated culturally responsive evaluation (CRE) (Hood et al., 2015), a framework for centering an evaluation in the culture of the programs being evaluated, with implementation and outcome constructs from the updated Consolidated Framework for Implementation Research (CFIR) (Damschroder et al., 2022). This integration would ensure that useful evidence was produced to inform implementation of diabetes interventions in real-world settings that reach at-risk populations. Fish et al. (2023) used the RE-AIM framework to evaluate the implementation of an organization- and therapist-focused training program adapted for virtual delivery due to COVID-19. The program aimed to improve mental health workforce’s cultural competence in working with the LGBTQ + community. Both studies aimed to better understand strategies for ensuring that EBPIs reached individuals from diverse groups that experience disparities in prevalence of disease and access to the best available programs.

## Conclusion

The purpose of the *Advancing the Adaptability of Chronic Disease Prevention and Management Through Implementation Science* special supplement is to examine and illustrate the value of implementation science in chronic disease prevention and management, sharing information to support infrastructure for the scale and spread of evidence-based interventions. Via the Effective Practice Pipeline, the Editors provide a new perspective on implementation science’s role in public health to help practitioners maximize public health impact. The articles in this supplement are a testament to how implementation science is necessary to support the Effective Practice Pipeline to improve practice in context for diverse populations.

The pandemic highlighted health disparities and access to care, shining a light on long-held disparities among those with chronic disease conditions. A decade and a half into the identified Implementation Science field, intervention research, implementation research, and implementation practice can no longer continue to advance in silos if a population impact is to be achieved. Maintaining these silos has the potential to overlook community need and capacity and increase health disparities. Known strategies (e.g., UNISIC activities) have been identified by implementation research to enhance the potential for interventions to successfully achieve targeted intervention and implementation outcomes, but implementation practitioners need to have access to how to use this knowledge under different contexts. Conversely, implementation practitioners have firsthand experience supporting a range of adopters and can provide unique insights to researchers about strategies and the mechanisms they activate. The integration of all three pipelines is a critical and necessary next step for the implementation science and chronic disease fields.

In addition, we cannot design and implement interventions to prevent chronic disease without better understanding context, our communities, and the “conditions in which people are born, grow, live, work, and age, and the wider set of forces and systems shaping the conditions of daily life” (World Health Organization Resolution WHA62.14, 2022; https://apps.who.int/gb/ebwha/pdf_files/WHA62-REC1/WHA62_REC1-en-P2.pdf). These factors can influence health outcomes. Implementation scientists have developed many frameworks, theories, and models (Tabak et al., 2012) which highlight the importance of context and the outer settings in which interventions are implemented. Relatedly, researchers cannot develop interventions that fail to consider the real-world human capital, financial, and other resource constraints of most public health practice entities. A greater emphasis on designing EBIs for the intended delivery context is critical for initial adoption and sustained delivery (Kwan et al., 2022). The use of implementation research by public health practitioners has the distinct potential to reduce health disparities with the intention of addressing health inequities in chronic disease.
