# Correction: Preventing and Managing Chronic Disease Through Implementation Science: Editor’s Introduction to the Supplemental Issue

**DOI:** 10.1007/s11121-024-01680-z

**Published:** 2024-05-15

**Authors:** Justin D. Smith, Sandra F. Naoom, Lisa Saldana, Sharada Shantharam, Tina Anderson Smith, Jennifer M. Kohr

**Affiliations:** 1 Department of Population Health Sciences, Division of Health Systems Innovation and Research, Spencer Fox Eccles School of Medicine at the University of Utah, 295 Chipeta Way, Salt Lake City, UT 84108 USA; 2 JSFB LLC, Northbrook, IL USA; 3 Chestnut Health Systems, Lighthouse Institute, Eugene, OR USA; 4 Centers for Disease Control and Prevention, Division for Heart Disease and Stroke Prevention, Atlanta, GA USA; 5 Anderson Smith Consulting, Atlanta, GA USA; 6 Centers for Disease Control and Prevention, Performance and Evaluation Office, Atlanta, GA USA

**Correction to: Prevention Science** 10.1007/s11121-023-01617-y

The article “Preventing and Managing Chronic Disease Through Implementation Science: Editor’s Introduction to the Supplemental Issue”, written by Smith, J.D., Naoom, S.F., Saldana, L., Shantharam, S., Anderson Smith, T., and Kohr, J.M., was originally published electronically on the publisher’s internet portal on 01 December 2023 without open access. With the author(s)’ decision to opt for Open Choice the copyright of the article changed on 12 January 2024 to © The Author(s) 2024 and the article is forthwith distributed under a Creative Commons Attribution 4.0 International License, which permits use, sharing, adaptation, distribution and reproduction in any medium or format, as long as you give appropriate credit to the original author(s) and the source, provide a link to the Creative Commons licence, and indicate if changes were made. The images or other third party material in this article are included in the article’s Creative Commons licence, unless indicated otherwise in a credit line to the material. If material is not included in the article’s Creative Commons licence and your intended use is not permitted by statutory regulation or exceeds the permitted use, you will need to obtain permission directly from the copyright holder. To view a copy of this licence, visit http://creativecommons.org/licenses/by/4.0/.

The original article has been corrected.

